# Light-Induced Metallic and Paramagnetic Defects in Halide Perovskites from Magnetic Resonance

**DOI:** 10.1021/acsenergylett.4c02557

**Published:** 2024-09-25

**Authors:** Aditya Mishra, Michael A. Hope, Lyndon Emsley

**Affiliations:** Institut des Sciences et Ingénierie Chimiques, Ecole Polytechnique Fédérale de Lausanne, Lausanne CH-1015, Switzerland

## Abstract

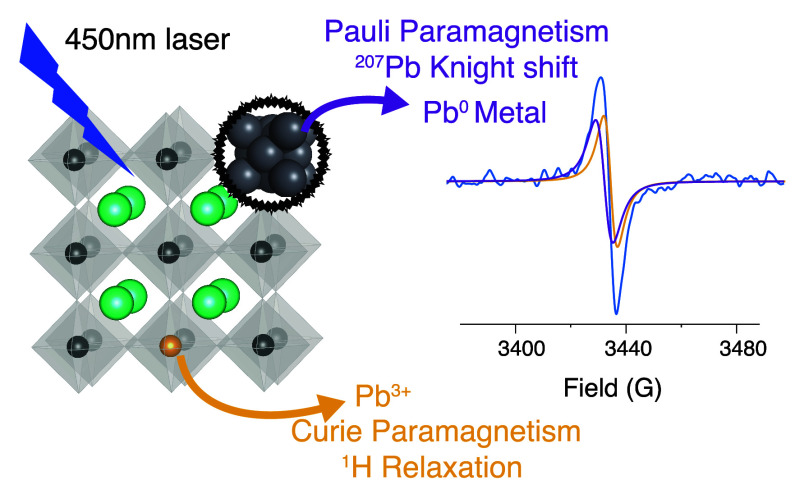

Halide perovskites are promising next-generation solar cell materials, but their commercialization is hampered by their propensity to degrade under operating conditions, particularly under heat, humidity, and light. Identifying degradation products and linking them to the degradation mechanism at the atomic scale is necessary to design more stable perovskite materials. Here we use magnetic resonance methods to identify and characterize the formation of both metallic lead clusters and Pb^3+^ defects upon light-induced degradation of methylammonium lead halide perovskite using nuclear magnetic resonance (NMR) and electron paramagnetic resonance (EPR) measurements. Paramagnetic relaxation enhancement (PRE) of the ^1^H NMR resonances demonstrates the presence of localized paramagnetic Pb^3+^ defects, a large Knight shift of the ^207^Pb NMR proves the presence of lead metal, and their relative proportions are determined by the differing temperature dependence in variable-temperature EPR. This work reconciles previous conflicting literature results, enabling the use of EPR spectroscopy to monitor photodegradation of perovskite devices.

Halide perovskites have gained immense interest in the past decade owing to their exceptional optoelectronic properties for use in optoelectronics devices, notably photovoltaics.^1−4^ Perovskite-based thin-film photovoltaics can already achieve power conversion efficiencies up to 26%.^5^ Perovskite materials adopt an ABX_3_ crystal structure (Figure 1), where A^+^ is a monovalent cation such as methylammonium (MA^+^), formamidinium (FA^+^), and/or cesium (Cs^+^), which resides in a cuboctahedral cavity formed by a three-dimensional network of [BX_6_]^4–^ corner-sharing octahedra, where B^2+^ is a bivalent cation such as lead (Pb^2+^) and tin (Sn^2+^), and X^–^ is a halogen ion (Cl^–^, Br^–^, or I^–^).^6^ These are inexpensive and solution-processable materials, with near-optimal band gaps, long charge-carrier diffusion lengths, and high absorption coefficients.^1−3^ As such, perovskite solar cells have the potential to deliver significantly cheaper solar energy.^7^

**Figure 1:**
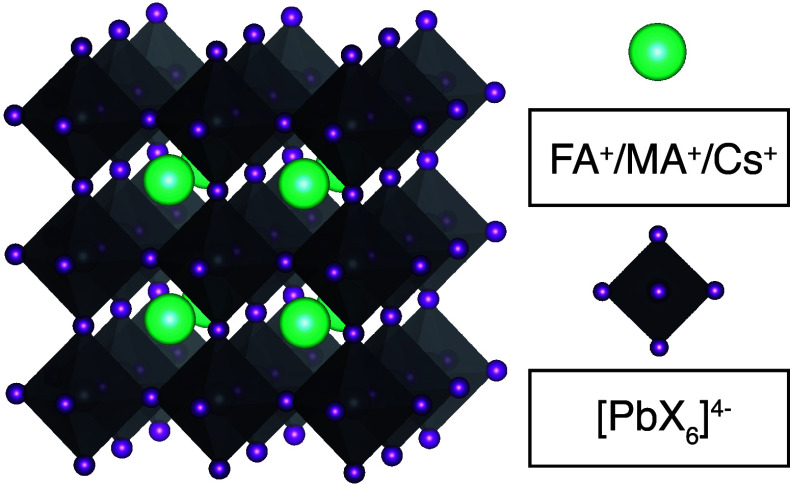
Schematic representation of the ABX_3_ perovskite lattice.

Despite possessing such promising optoelectronic properties, degradation induced by external stimuli such as heat, humidity, electric bias, and light hinders their commercial application.^8−13^ Among these, light-driven degradation of the perovskite absorber layer under operating conditions poses a major bottleneck.^9^ One of the major light-induced degradation mechanisms is the generation of metallic lead (Pb^0^) species under continuous light irradiation.^14−25^ This has primarily been observed using X-ray photoelectron spectroscopy (XPS) measurements, although these are limited to observation of the surface.^26^ On extended degradation, volatile species such as ammonia (NH_3_), aminocarbyne fragments (CNH_2_), hydrogen (H_2_), and iodine/hydrogen iodide (I_2_/HI) are evolved, that have been detected utilizing in situ mass-spectrometry.^27^ The initial stages of photodegradation have been studied for MAPbI_3_, proving that photoinstability can occur via the formation of point defects including iodide vacancies, iodide interstitials, and Pb^3+^ centers which act as charge-carrier traps.^28^ The presence of these light-induced defects are usually inferred from their effect on the optoelectronic properties via optical and electrical measurements, including monitoring changes in the photoluminescence, absorption/emission, and electroluminescence under light irradiation.^14,16,29−40^ These methods provide indirect evidence for defect formation, but direct atomic-scale measurements are required to develop structure–activity relationships for these materials.

Magnetic resonance methods, including nuclear magnetic resonance (NMR) and electron paramagnetic resonance (EPR) spectroscopies, are nondestructive analytical probes of atomic-level structure.^41,42^ NMR has been actively used within the perovskite photovoltaics community for surface^43−45^ and bulk perovskite characterization.^46−55^ The focus of NMR spectroscopy is typically diamagnetic materials, although NMR of paramagnetic materials can also reveal a wealth of information.^56,57^ EPR spectroscopy, on the other hand, directly probes the unpaired electrons in paramagnetic materials.^42,58^ Primarily EPR is used to study localized radicals, which exhibit Curie paramagnetism.^42,59^ However, metals also possess unpaired electrons due to Pauli paramagnetism of the delocalized free electrons and, therefore, metals can also be identified using EPR (this is sometimes known as conduction electron spin resonance, CESR).^60−62^ The Pauli paramagnetic electrons also couple to nuclear spins, resulting in very large chemical shifts in NMR spectra known as Knight shifts.^63^ These signals are well outside the range of diamagnetic shifts and can be readily used to identify metallic species.^64,65^

EPR has previously been applied to study hybrid perovskites.^66−69^ Shkrob and Marin observed the formation of ^•^CH_2_NH_3_ after irradiation of MAPbBr_3_ with 3 MeV electrons at 50 K, with a multiplet structure due to hyperfine coupling with ^1^H and ^14^N.^69^ They also studied irradiation of MAPbBr_3_ with a 355 nm laser at 50 K, reporting a broad signal around *g* = 2 that they ascribe to lead metal, and a minor sharp component ascribed to localized Pb^3+^ centers. In contrast, Collela et al. illuminated MAPbI_3_ at room temperature with a tungsten lamp and ascribed the signal around *g* = 2 to Pb^3+^.^68^ This inconsistency in the literature leads to ambiguity in interpreting the results of EPR measurements.

Here we identify the signatures of both lead metal clusters and Pb^3+^ centers following illumination of MAPbI_3_ and MAPbI_1.5_Br_1.5_, by combining variable-temperature EPR, ^1^H NMR relaxation, and ^207^Pb NMR experiments. Quantification of the EPR spectra yields around 40 metallic lead atoms per Pb^3+^ defect in the MAPbI_3_ sample studied here. This reconciles previous literature studies and demonstrates the importance of both species when interpreting perovskite photodegradation results.

In order to observe light-induced paramagnetic species in bulk mechanosynthsized MAPbI_3_ perovskite, room-temperature EPR measurements were taken before and after ex situ irradiation of a capillary of bulk MAPbI_3_ powder with a continuous wave 450 nm laser (Figure 2). There are no significant paramagnetic impurities in the pristine diamagnetic MAPbI_3_ material as expected, whereas light irradiation induces a clear signal, indicating the generation of paramagnetic species. This EPR signal was consistently reproducible in other MAPbI_3_ samples (Figure S1) or in a mixed halide composition (Figure S2). The observed line widths were the same for MAPbI_3_ and MAPbBr_1.5_I_1.5_ (Figure S3), suggesting that the line width is not dominated by hyperfine coupling to ^127^I or ^79/81^Br.

**Figure 2:**
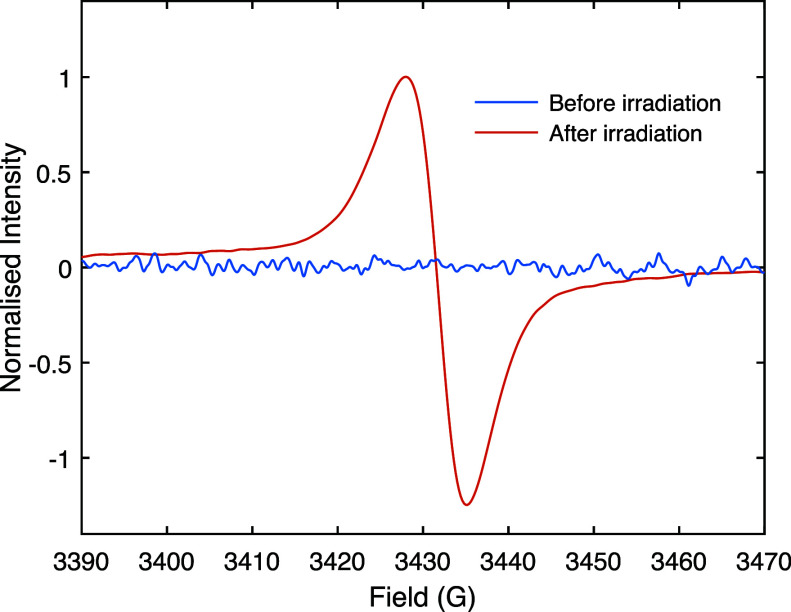
Experimental X-band continuous wave EPR spectra of MAPbI_3_ at room temperature before and after laser irradiation for 120 s with a 1.5 W/cm^2^ laser operating at 450 nm. The greater noise in the spectrum acquired before irradiation is due to fewer transients being acquired (4 vs 64) and a lower receiver gain (50 dB vs 58 dB). Further parameters are given in Table S2.

We now consider the origin of the paramagnetic species. The signal we observe here does not exhibit any fine structure from hyperfine coupling to magnetically active ^1^H and ^14^N nuclear spins of the MA^+^ ion, which rules out a cation-centered radical. Pb^3+^ radicals generated by irradiation of various lead salts have previously been reported with *g* factors in the range 2.007–2.034 (Table S1),^68,70,71^ while EPR of photogenerated Pb^0^ metal clusters has previously been reported with *g* factors in the range 2.0009–2.0054 (Table S1),^72^ although these studies were all performed at low temperatures of 50–110 K. The observed average *g* factor here of 2.0045 is more consistent with the reported range for lead metal. The EPR spectrum of bulk lead metal is broadened beyond detection due to rapid relaxation; therefore, it is only possible to observe particles smaller than ca. 100 nm, where quantum confinement effects suppress relaxation.^73,74^ Consequently, it was not possible to obtain a reference spectrum of pure lead metal (i.e., not photogenerated), and to our knowledge, this has not been achieved in the literature.

In order to distinguish between Pb^3+^ radicals and lead metal clusters, we performed variable-temperature (VT) EPR measurements. Localized paramagnetic defects exhibit Curie paramagnetism, meaning that the magnetization increases with decreasing temperature, following a 1/*T* dependence. In contrast, the Pauli paramagnetism of delocalized metallic electrons is temperature independent.^65^Figure 3a shows the VT EPR spectra of MAPbI_3_ after light illumination at room temperature. The signal intensity increases with decreasing temperature, suggesting a localized Pb^3+^ defect. However, the double integration of the VT EPR spectra cannot be fit with a simple Curie 1/*T* dependence (Figure 3b). Including a temperature-independent Pauli contribution successfully reproduces the experimental data (Figure 3b, Figure S4). At room temperature, the Pauli component contributes 58% of the signal, highlighting the importance of considering both species. This corresponds to a molar ratio of 41 metallic lead atoms per Pb^3+^ defect, see Supplementary Note 1 for details.

**Figure 3:**
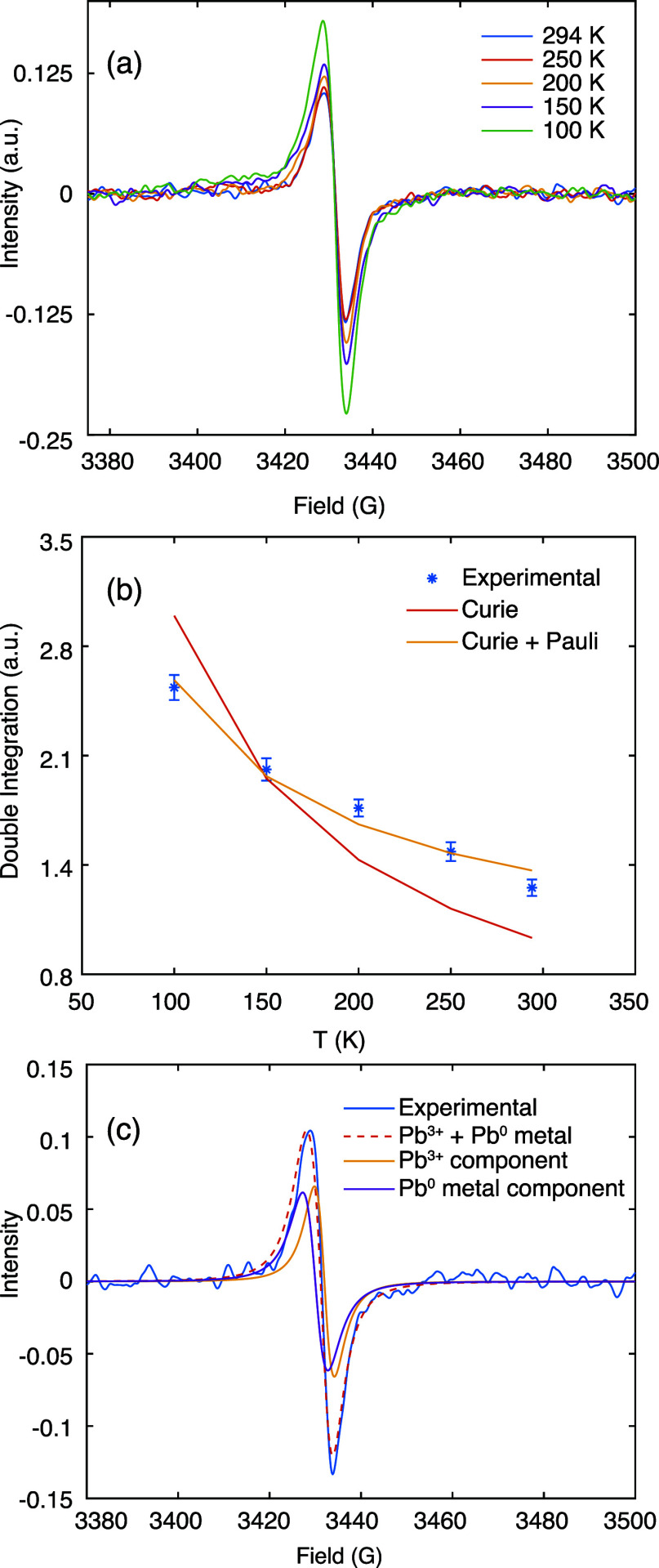
(a) Experimental X-band continuous wave EPR spectra of MAPbI_3_ as a function of temperature after ex situ laser irradiation for 120 s with an ∼1.5 W laser operating at 450 nm. (b) Double integration of the VT EPR spectra as a function of temperature, scaled by the measured *Q* factor at each temperature (Table S3). The error bars were calculated assuming that spectral noise dominates the uncertainty in the double integrals, but other sources may also contribute, such as the measured *Q* factors. (c) Deconvolution of the room-temperature EPR spectrum into the Curie (Pb^3+^) and Pauli (lead metal) components. The optimized broadening parameters for both components are listed in Table S4. Further details are presented in the Experimental Section.

With this knowledge, the EPR spectra can be deconvoluted into the Pauli and Curie components (Figures S5 and S6). The VT EPR spectra were simultaneously fitted at all temperatures, with intensities constrained to give double integrations in the ratio determined by a Curie/Pauli temperature dependence analysis. The different temperature dependence of the Curie and Pauli signals result in different relative contributions at each temperature, resolving the ambiguity of deconvoluting the broad signal. Figure 3 shows the deconvolution of both components at room temperature with *g*-factors of 2.0038 for Pb^3+^ (Curie component) and 2.0050 for lead metal (Pauli component). The *g*-value obtained here for Pb metal is consistent with previously reported ranges (Table S1), and the value for Pb^3+^ is close to that observed by Colella et al.^68^ Note that the EPR spectrum cannot be satisfactorily fit with a single Gaussian–Lorentzian line (Figure S7). These *g*-values can also reproduce the spectra obtained from three different samples with different ratios of Pb^3+^ and Pb metal (Figure S1), further supporting the deconvolution.

To prove the presence of both Pb^3+^ defects and lead metal clusters, we conducted NMR measurements. Figure 4a shows the ^207^Pb spectrum of a reference sample of metallic lead with a single resonance at ∼11100 ppm. This huge chemical shift, well outside the diamagnetic shift range (−5500 to 6000 ppm) is the Knight shift arising from coupling of the conduction electrons with the ^207^Pb nuclei in lead metal.^75^Figure 4b shows the ^207^Pb spectrum of MAPbI_3_ after laser illumination, which clearly shows a signal from metallic lead. The signal is weak owing to the low sample mass (∼6 mg) and the fact that the concentration of lead metal in the sample is very low.

**Figure 4:**
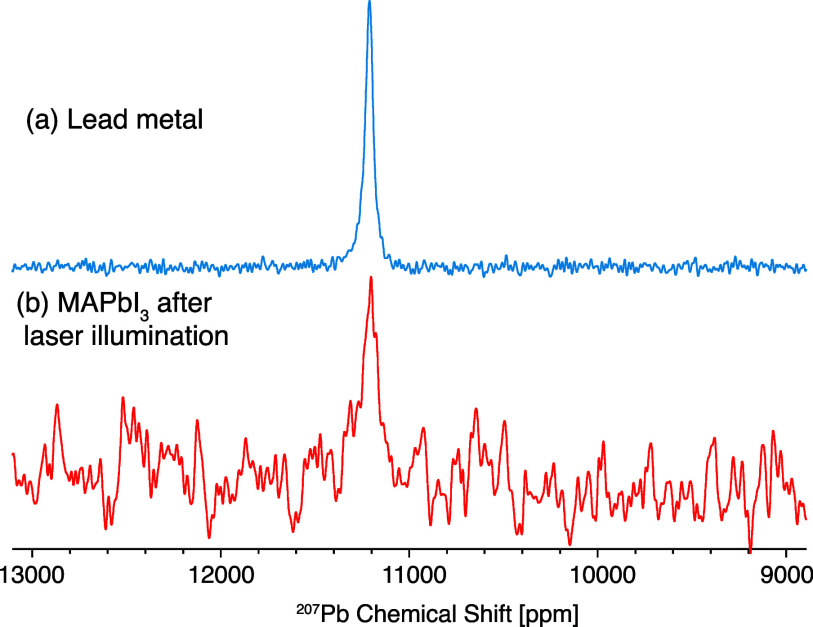
Echo-detected ^207^Pb MAS NMR spectra of (a) lead metal and (b) MAPbI_3_ after laser illumination.

To confirm the presence of Pb^3+^ radicals after light illumination, ^1^H relaxation measurements of MAPbI_3_ perovskite were conducted. Pb^3+^ is paramagnetic and, therefore, cannot typically be observed directly by ^207^Pb NMR. It does, however, induce paramagnetic relaxation enhancement (PRE) of the diamagnetic ^1^H nuclei in the organic cation. The rapidly fluctuating unpaired electron spin induces fast relaxation of dipolar-coupled nearby ^1^H spins; ^1^H–^1^H spin diffusion then acts to equilibrate the relaxation within the sample, leading to a reduction in the average *T*_1_ relaxation time. PRE has previously been used to demonstrate the incorporation and distribution of paramagnetic dopants in the CsPbX_3_ perovskite lattice, for instance.^57,76^

Figure 5 shows a light-induced effect on the nuclear spin relaxation times (*T*_1_) of ^1^H nuclei in MAPbI_3_ perovskite (Table 1). Before light irradiation, both ammonium (−NH_3_) and methyl (−CH_3_) protons in MA^+^ relax with a time constant of 14.1 s. After light irradiation, the relaxation time constants for −NH_3_ and −CH_3_ are reduced to 9 and 7 s, respectively, which shows a relaxation enhancement of ^1^H from photogenerated paramagnetic species following light irradiation. The stretching parameters of β ≈ 0.87 indicate a small distribution of *T*_1_ in the material, due to a distribution of Pb^3+^ concentrations. The PRE results corroborate the presence of photogenerated Pb^3+^ paramagnetic species in the material (although we cannot rule out the presence of other EPR-silent paramagnetic species). A similar PRE analysis was also performed for the mixed-halide perovskite composition (MAPbI_1.5_Br_1.5_) and the same trend was observed (Figure S8 and Table S5).

**Figure 5:**
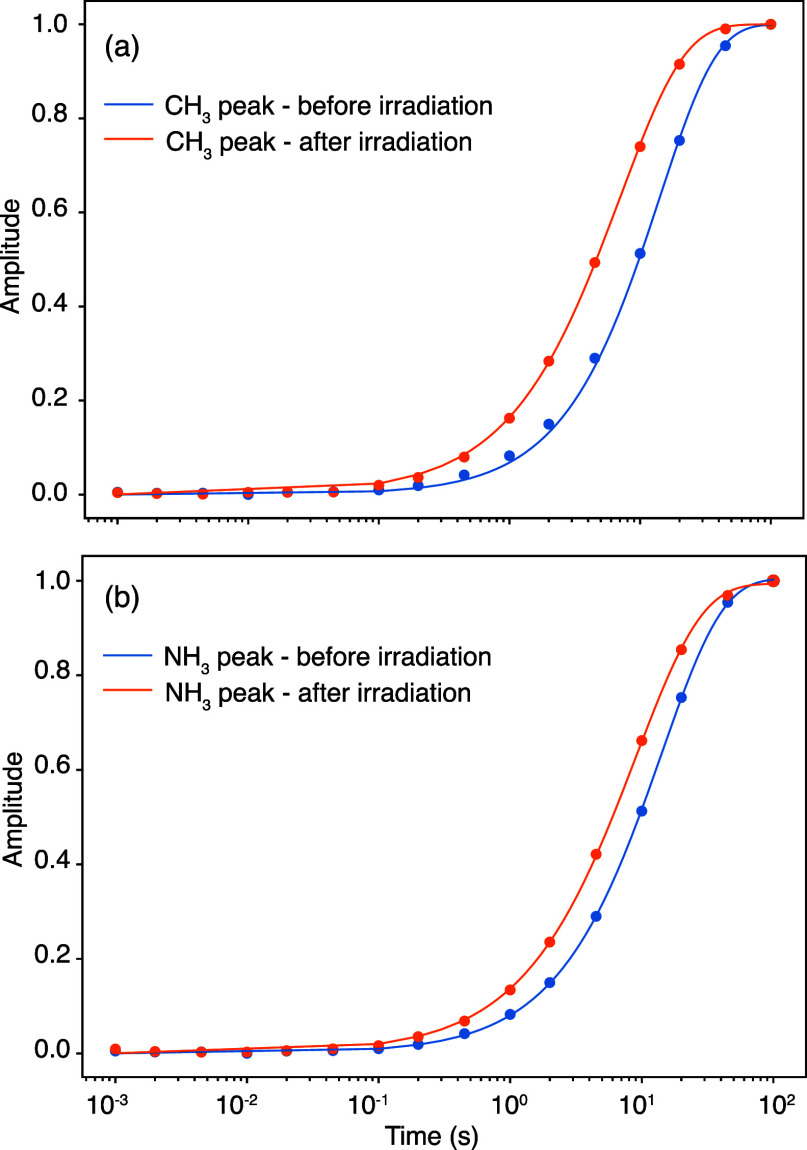
Light-induced changes in ^1^H relaxation times in the MAPbI_3_ perovskite. Experimental data are shown with circles, and the solid lines are fits to a stretched exponential function, *I* = *I*_0_ (1 – exp[−(*t*/*T*_1_)^β^]), with the parameters in Table 1. Further details are presented in the Experimental Section.

**Table 1:** Effect of Light Irradiation on *T*_1_ Relaxation Times in MAPbI_3_

	–NH_3_		–CH_3_	
	*T*_1_/s	β	*T*_1_/s	β
before irradiation	14.1 ± 0.3	0.93	14.1 ± 0.3	1.00
after irradiation	9.0 ± 0.1	0.86	7.0 ± 0.1	0.88

We have presented a combined NMR and EPR study of halide perovskite degradation under light. Using ^207^Pb NMR and ^1^H *T*_1_ measurements, we unambiguously demonstrate that both Pb^0^ metal clusters and paramagnetic defects are present after the irradiation of MAPbI_3_. The temperature dependence of the EPR signal in these samples indicates that it comprises overlapping signals from both Pb^3+^ defects and Pb^0^ metal, which can be deconvoluted and quantified. This reconciles the conflicting assignment of this signal in previous literature^68,69^ and demonstrates that both species should be considered when interpreting the EPR spectra of photodegraded lead systems. Notably, under the conditions used here, we find that after light irradiation the large majority of lead impurities are present as metallic lead, with around 40 metallic lead atoms per Pb^3+^ defect.

## Experimental Section

### Materials

The following materials were used without further purification: methylammonium iodide (Sigma, 99.9%), lead iodide (Sigma, 99.9%), and lead metal powder (Alfa Aesar, 99.95%, 100 mesh, <150 μm).

### Bulk Sample Preparation

The materials were prepared using mechanosynthesis following a previously published protocol.^77^ The precursors (MAI and PbI_2_) were mixed in the appropriate molar ratio and ground in an electric ball mill (Retsch MM 400) using an Eppendorf jar (10 mL) and steel ball (*⌀* 10 mm) for 60 min at 25 Hz.

### Light Irradiation

Ex situ illumination experiments were performed by packing MAPbI_3_ into a 1.8 mm outer-diameter quartz capillary and exposing it to a continuous-wave 450 nm laser with an output power of ∼2 W cm^–2^.

### EPR Measurements

EPR spectra were measured using a Bruker EMX nano X-band spectrometer using either 0.2 or 0.4 mT modulation amplitude. The EasySpin suite in Matlab was used for background subtraction, deconvolution, and fitting.^78^ The variable-temperature EPR spectra were simultaneously fitted using Matlab with two Gaussian–Lorentzian signals, corresponding to the Pauli (Pb metal) and Curie (Pb^3+^) components. For each component, the *g*-factor, Lorentzian broadening, and Gaussian broadening were refined but kept the same for each temperature. The ratio of the two components was fixed at each temperature by assuming that the Pauli paramagnetism is temperature independent, and the Curie paramagnetism follows a 1/*T* dependence. An overall scaling parameter was refined at each temperature, giving a total of 11 fitting parameters.

### Solid-State NMR Measurements

NMR experiments were performed on a commercial Bruker Avance III 500 MHz (11.7 T) NMR spectrometer using a 3.2 mm triple resonance low-temperature magic angle spinning (LTMAS) probe with zirconia rotors spinning at 10 kHz. Room-temperature ^207^Pb spectra were acquired with a Hahn echo with a repetition time of 4.3 ms. For NMR of Pb metal, Pb metal powder was diluted with KBr 1:1 by mass. The ex situ irradiated MAPbI_3_ sample was unpacked from the capillary for the NMR measurements. For lead metal and irradiated MAPbI_3_, 3200 and 12.8 million transients were collected, respectively. ^207^Pb chemical shifts were referenced to the room-temperature peak of lead nitrate at −3492 ppm. PRE NMR experiments were performed on a commercial Bruker Avance III 400 MHz (9.4 T) NMR spectrometer using a 3.2 mm triple-resonance low-temperature magic angle spinning (LTMAS) probe with zirconia rotors spinning at 15 kHz. Room-temperature ^1^H relaxation times were measured with saturation recovery experiments and fitted to a stretched exponential function, *I* = *I*_0_(1 – exp[−(*t*/*T*_1_)^β^]). Errors were estimated by Monte Carlo analysis.

## Data Availability

All data presented here (raw NMR and EPR data, and Matlab scripts) can be accessed at the link DOI: 10.5281/zenodo.13830953 and is available under the CC-BY-4.0 (Creative Commons Attribution-ShareAlike 4.0 International) license.

## Supporting Information Available

Additional EPR and NMR data (PDF)
