# Supplementary material for: Light-Induced Metallic and Paramagnetic Defects in Halide Perovskites from Magnetic Resonance
Source: ACS Energy Lett. 2024 Sep 25;9(10):5074–80. doi: 10.1021/acsenergylett.4c02557 (PMC11474947; doi:10.1021/acsenergylett.4c02557)
Supplement: Supplementary file 1 — nz4c02557_si_001.pdf [file nz4c02557_si_001.pdf]

Supporting information

Light-Induced Metallic and Paramagnetic Defects in Halide Perovskites from Magnetic Resonance

*Aditya Mishra, Michael A. Hope, Lyndon Emsley**

Institut des Sciences et Ingénierie Chimiques, Ecole Polytechnique Fédérale de Lausanne, Lausanne CH – 1015, Switzerland

Email correspondance: lyndon.emsley@epfl.ch

Raw Data. All the raw data associated with the manuscript can be accessed at the following link DOI: 10.5281/zenodo.13830953 and is available under the CC-BY-4.0 (Creative Commons Attribution-ShareAlike 4.0 International) license.

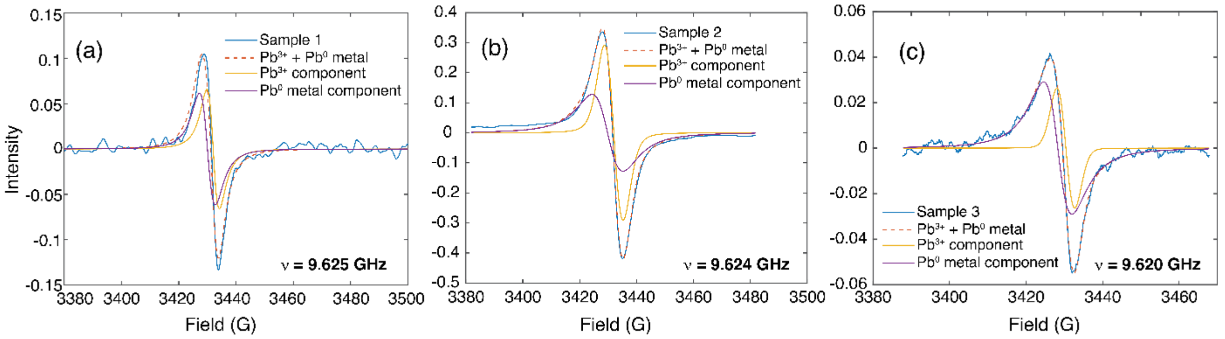

Figure S1. Experimental X-band continuous wave EPR spectra of three different $MAPbI_3$ samples measured at room temperature after laser irradiation. Each spectrum is deconvoluted with two components, Pb^0 with g = 2.0049893 and Pb^{3+} with g = 2.0037745, but with different relative contributions. The deconvolutions successfully reproduce the observed spectra for all three samples.

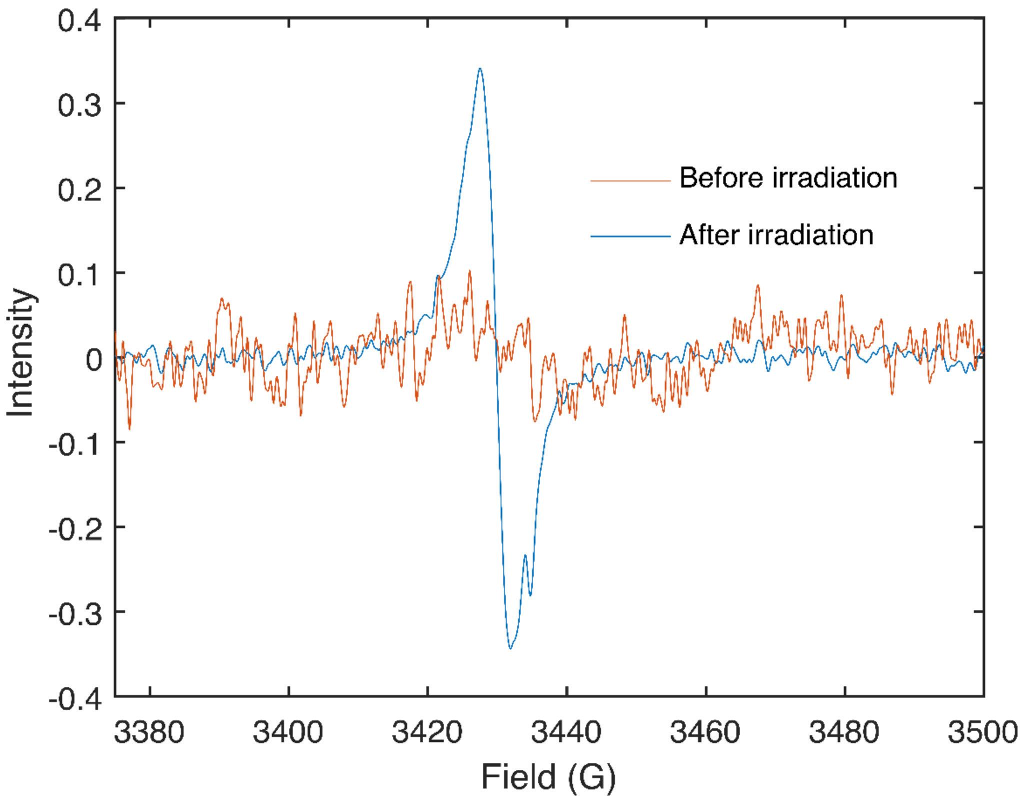

Figure S2. Experimental X-band continuous wave EPR spectrum of $MAPbBr_{1.5}I_{1.5}$ sample acquired before and after *ex-situ* laser irradiation at 450 nm at room-temperature. Experimental parameters are given in Table S2.

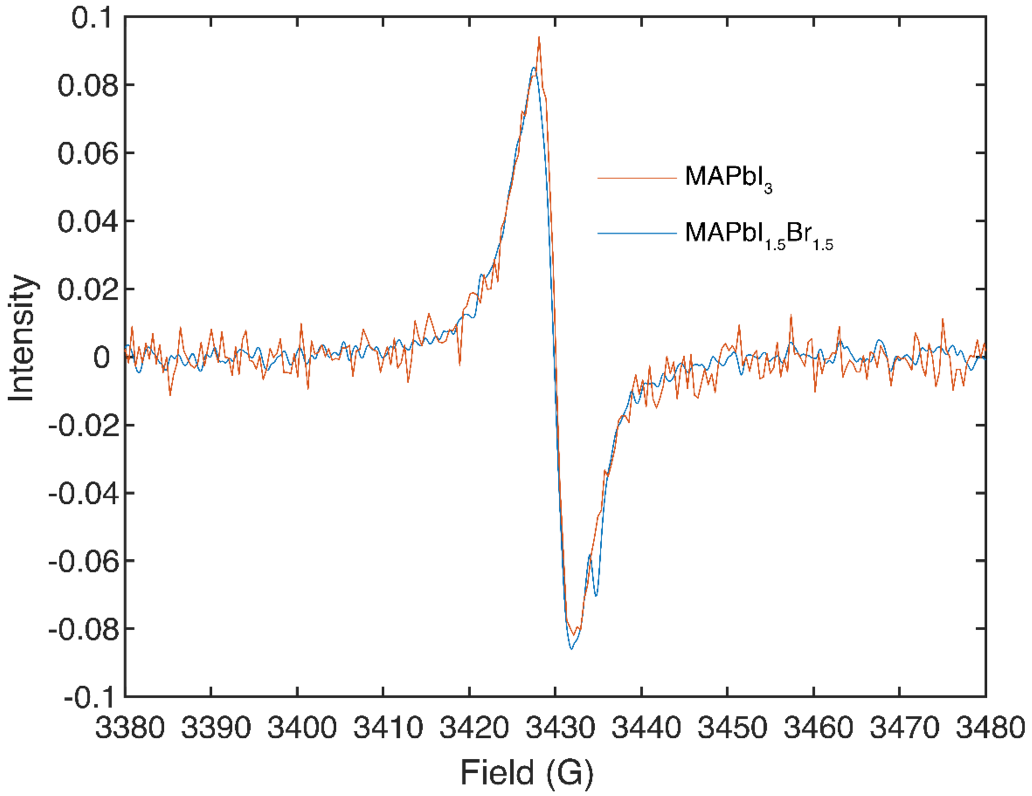

Figure S3. Comparison of experimentally measured X-band EPR spectra for pure iodide and mixed-halide samples. The comparable EPR linewidths suggests a negligible contribution from hyperfine coupling to ^{127}I and/or $^{79/81}$Br.

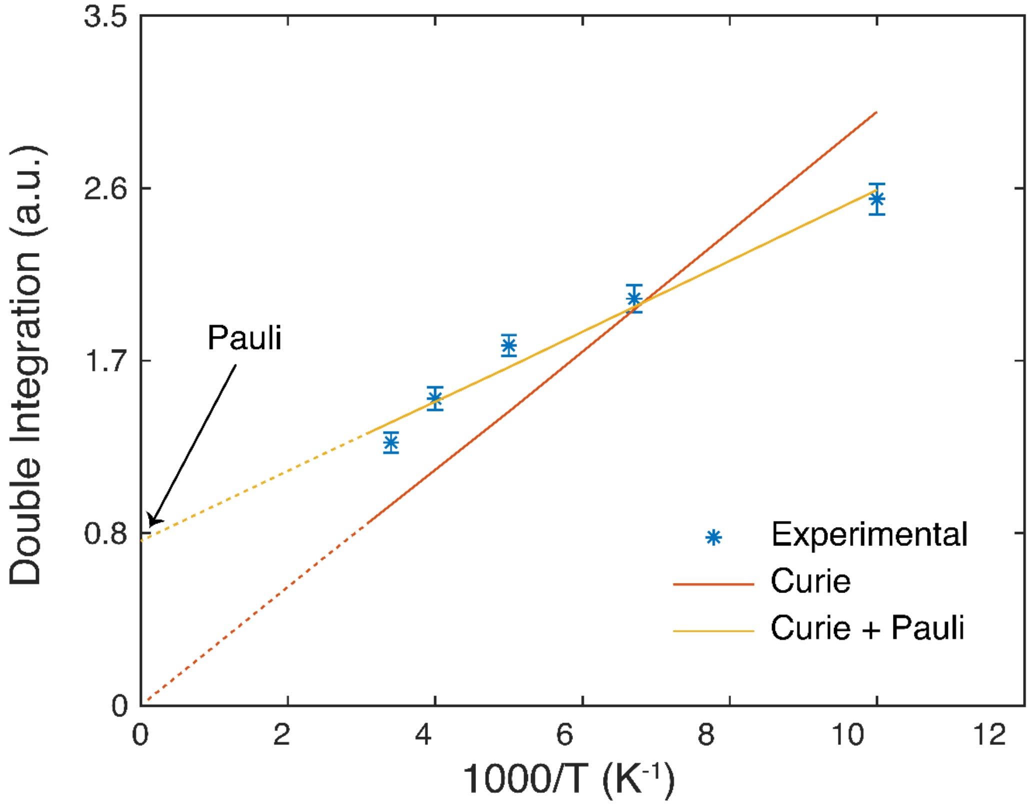

Figure S4. Double integration of the light-irradiated $MAPbI_3$ VT EPR spectra as a function of inverse temperature. In this fit, the y-axis intercept represents the temperature-independent Pauli contribution.

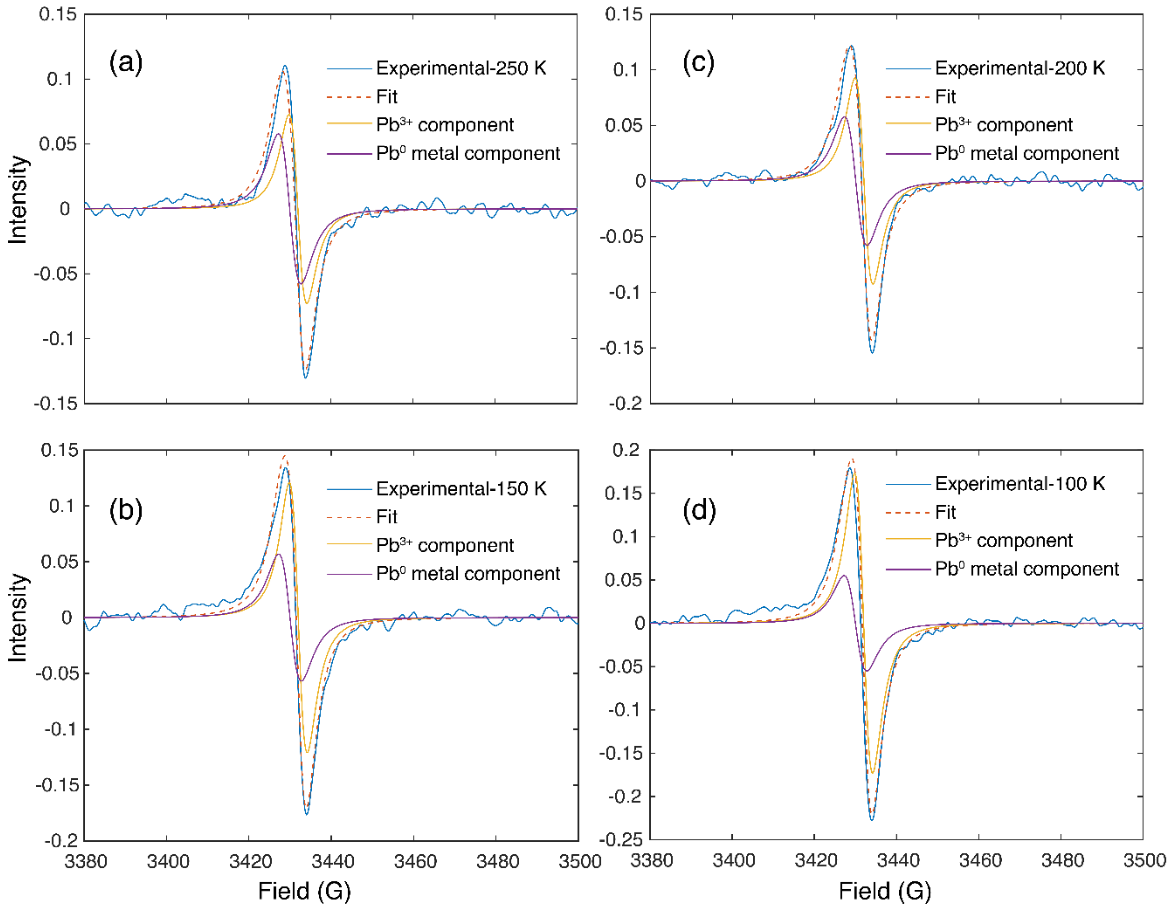

Figure S5. Fitting of the variable temperature EPR spectra for light-irradiated $MAPbI_3$ along with deconvolution into Pb^{3+} and Pb^0 metal components. The optimized g-values for the two components are 2.0038 and 2.0050, respectively.

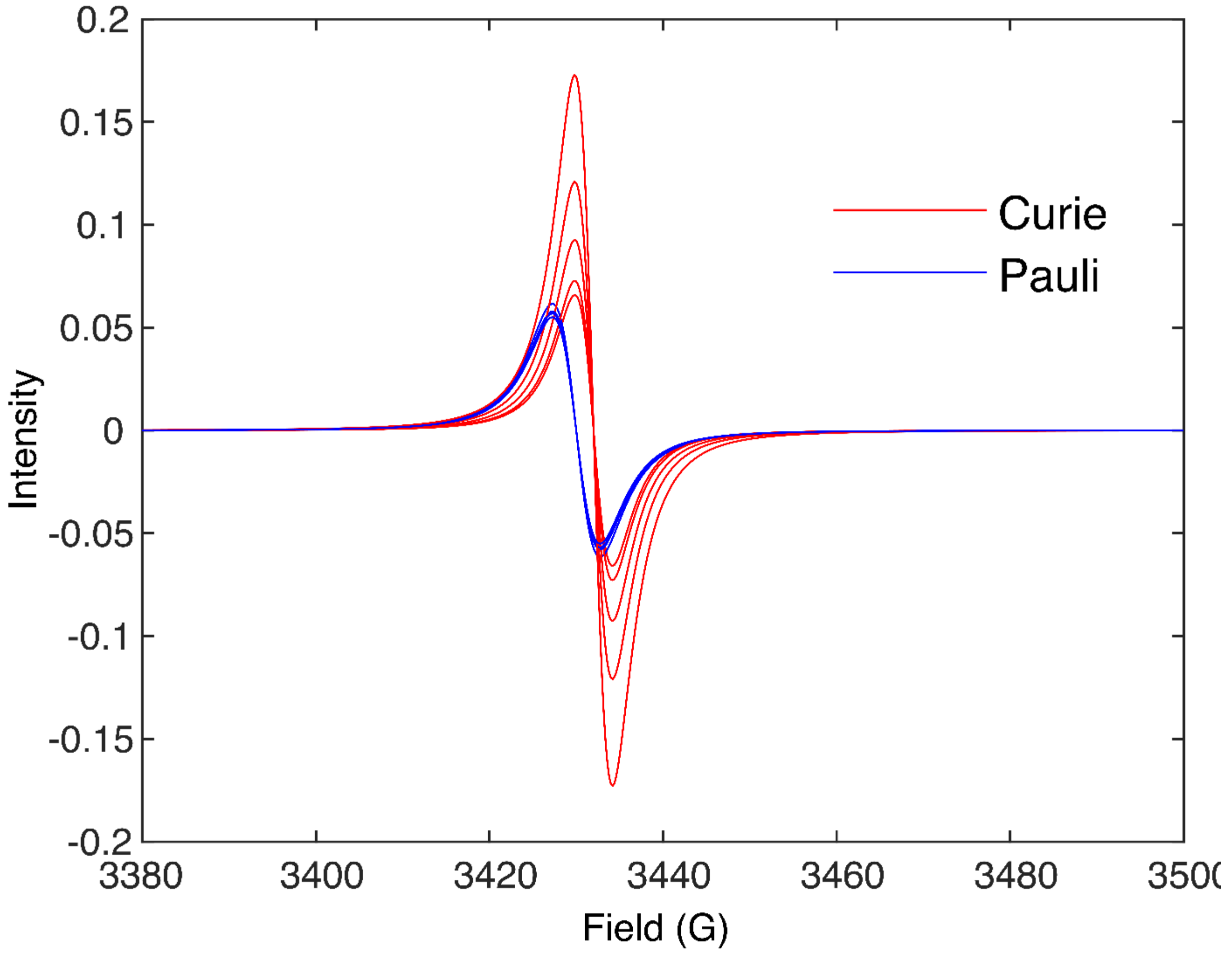

Figure S6. Curie (Pb^{3+}) and Pauli (Pb^{0}) components of the EPR spectra for light-irradiated $MAPbI_3$ from 100 – 294 K.

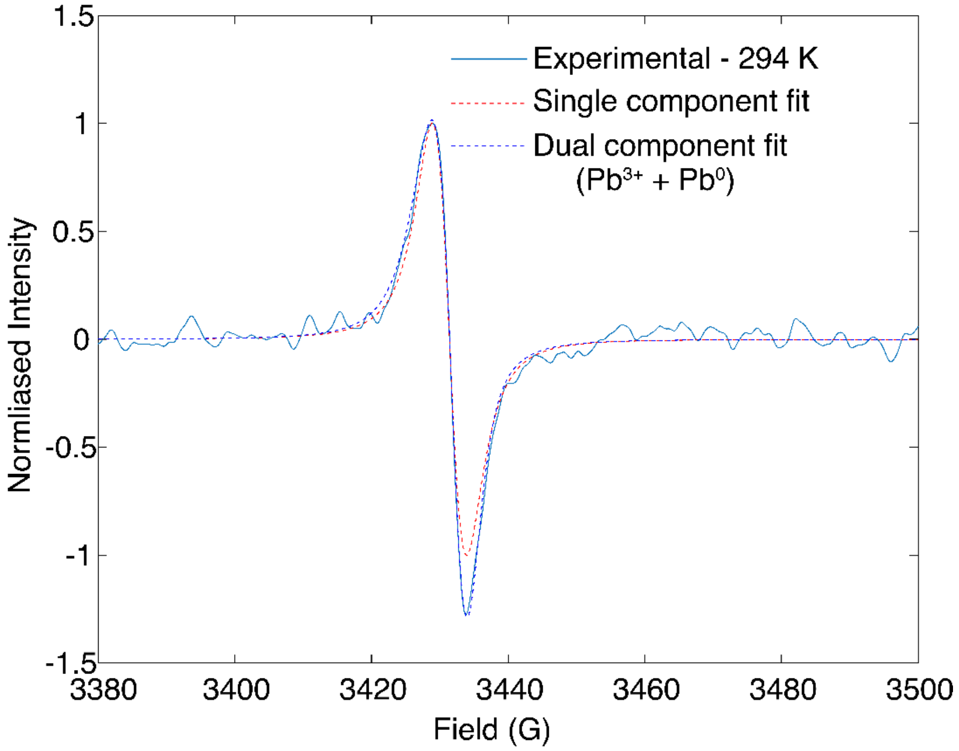

Figure S7. Fitting of the RT EPR spectrum for light-irradiated $MAPbI_3$ with either one or two Gaussian–Lorentzian functions, each defined by a g-value (*g*), Lorentzian broadening (L), and Gaussian broadening (G). For the single component fit, g = 2.0040, L = 0.2 mT, and G = 0.4 mT. In the dual component fit, the Curie component has g = 2.0038, L = 0.2 mT, and G = 0.2 mT, and the Pauli component has g = 2.0050, L = 0.2 mT, and G = 0.5 mT.

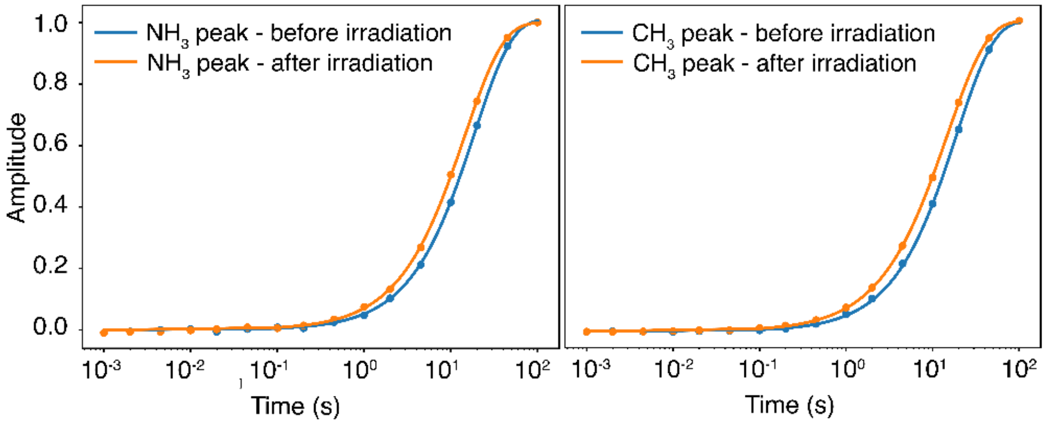

Figure S8. [1]H saturation-recovery curves measured before and after irradiation of $MAPbI_{1.5}Br_{1.5}$ bulk perovskite. Experimental data are shown with circles and the solid lines are fits to a stretched exponential function, $I = I_0\left(1 - \exp\left[-(t/T_1)^{\beta}\right]\right)$, with the parameters in Table S5.

Supplementary Note 1. Pb^0/Pb^{3+} quantification

The double integrations of the Curie and Pauli signals in the EPR spectra depend on the molar paramagnetic susceptibilities (χ_m) of lead metal and Pb^{3+} spins, and their concentrations in the sample (n):

$$I_{\text{Curie}}(T) \propto n_{\text{Curie}}\, \chi_m^{\text{Curie}}(T)$$

$$I_{\text{Pauli}} \propto n_{\text{Pauli}}\, \chi_m^{\text{Pauli}}$$

The molar Pauli paramagnetism of lead metal is known in the literature from electronic heat capacity measurements:[1]

$$\chi_m^{\text{Pauli}} = 43 \times 10^{-6}\ \text{emu mol}^{-1} = 5.4 \times 10^{-10}\ \text{m}^3\ \text{mol}^{-1}.$$

The molar paramagnetism of Pb^{3+} can be calculated from Curie's law:

$$\chi_m^{\text{Curie}}(T) = \frac{N_{\text{A}}\, g^2\, \mu_0 \mu_B^2\, S(S+1)}{3 k_{\text{B}} T},$$

where N_{A} is Avogadro's constant, g is the electron g-factor, μ_0 is the permeability of free space, k_{B} is Boltzmann's constant, and the electron spin $S = 1/2$. At room temperature, for Pb^{3+} with $g = 2.0038$, this gives

$$\chi_m^{\text{Curie}}(294\ \text{K}) = 1.61 \times 10^{-8}\ \text{m}^3\ \text{mol}^{-1}.$$

In other words, at 294 K, Pb^{3+} ions are ~30 times more paramagnetic than lead metal, per lead ion.

At 294 K, the experimental ratio of the double integrations is

$$I_{\text{Pauli}}/I_{\text{Curie}} = 1.38.$$

Therefore, the ratio of lead metal atoms to Pb^{3+} ions is

$$\frac{n_{\text{Pauli}}}{n_{\text{Curie}}} = \frac{I_{\text{Pauli}}}{I_{\text{Curie}}(294\ K)} \frac{\chi_m^{\text{Curie}}(294\ K)}{\chi_m^{\text{Pauli}}} = 1.38 \times 30 = 41.$$

i.e., for each Pb^{3+} ion, we have 41 lead atoms in lead metal clusters.

Note that any error in χ_m^{Pauli}, for instance due to difficulty in converting from electronic heat capacity,[1] will propagate in this estimate.

Table S1. g-factors reported in the literature for metallic and paramagnetic Pb species.

Irradiation	Sample	Reported Species	Temperature (K)	g-factor	References
450 nm laser	$MAPbI_3$	Pb^0<br>Pb^{3+}	294	2.0050<br>2.0038	This work
UV lamp	PbI_2	Pb^0	80	2.0009	8
UV laser	$MAPbBr_3$	Pb^0	50	2.0054	9
UV lamp	$PbCl_2/PbBr_2$	Pb^{3+}	80	2.03	8
3 MeV e^-	$PbBr_2/PbI_2$	Pb^{3+}	50	2.008	9
3 MeV e^-	$MAPbBr_3$	Pb^{3+}	50	2.008	9
γ rays	Pb acetate	Pb^{3+}	107	2.007	10
X or γ ray	Pb/KCl	Pb^{3+}	80	2.034	11
Tungsten lamp	$MAPbI_3$	Pb^{3+}	300	2.0034	12

Table S2. Experimental parameters for EPR data used in this work.

**Parameters**	**Before irradiation (Figure 2)**	**After irradiation (Figure 2)**	**Before irradiation (Figure S2)**	**After irradiation (Figure S2)**
Frequency/GHz	9.625217	9.624998	9.618734	9.616339
Gain/dB	50	58	10	30
Scans	4	64	1	4
Power/mW	0.3162	3.981	0.3162	0.3162
Scan width/G	200	100	200	200
Modulation amplitude/G	1	2	1	1

Table S3. Temperature dependence of Q-factor

Temperature (K)	Q-factor
100	4909
150	4909
200	4090
250	4090
294	4908

Table S4. Fitting parameters used for deconvolution of the variable temperature EPR spectra of $MAPbI_3$ following light irradiation.

Component	g-value	Broadening (mT)	
		Lorentzian	Gaussian
Curie (Pb^{3+})	2.0037745	0.0812	0.4190
Pauli (Pb^{0})	2.0049893	0.1758	0.4943

Table S5 Effect of light irradiation on [1]H T_1 relaxation times in $MAPbI_{1.5}Br_{1.5}$

	$-NH_3$		$-CH_3$	
	T_1 /s	β	T_1 /s	β
Before irradiation	18.8 ± 0.3	1.0	19.3 ± 0.3	0.97
After irradiation	14.5 ± 0.1	0.97	14.8 ± 0.1	0.94

Supplementary References

1. The knight shift. In *Prog. Mater. Sci.*, Elsevier Science: 1976; Vol. 20, pp 3–21.
2. Hughes, A. E.; Jain, S. C., Metal colloids in ionic crystals. *Adv. Phys.* **1979,** *28* (6), 717–828.
3. Feher, G.; Kip, A. F., Electron Spin Resonance Absorption in Metals. I. Experimental. *Phys. Rev.* **1955,** *98* (2), 337–348.
4. Dyson, F. J., Electron Spin Resonance Absorption in Metals. II. Theory of Electron Diffusion and the Skin Effect. *Phys. Rev.* **1955,** *98* (2), 349–359.
5. Ashcroft, N. W.; Mermin, N. D., *Solid state physics*. Cengage Learning: 2022.
6. *CRC Handbook of Chemistry and Physics*. 104th ed.; CRC Press: 2023.
7. Appendix A: Physical and Mathematical Properties. In *Foundations for Microstrip Circuit Design*, 2016; pp 629–634.
8. Arends, J.; Verwey, J. F., ESR on UV Irradiated Lead Halides at 80° K. *physica status solidi (b)* **1967,** *23* (1), 137–145.
9. Shkrob, I. A.; Marin, T. W., Charge Trapping in Photovoltaically Active Perovskites and Related Halogenoplumbate Compounds. *J. Phys. Chem. Lett.* **2014,** *5* (7), 1066–1071.
10. Morton, J. R.; Preston, K. F.; Strach, S. J., Electron paramagnetic resonance spectra of .gamma.-irradiated germanium, tin, and lead tetraacetates. *J. Phys. Chem.* **1979,** *83* (7), 853–855.
11. Schoemaker, D.; Kolopus, J. L., Pb^{++} as a hole trap in KCl: ESR and optical absorption of Pb^{+++}. *Solid State Commun.* **1970,** *8* (6), 435–439.
12. Colella, S.; Todaro, M.; Masi, S.; Listorti, A.; Altamura, D.; Caliandro, R.; Giannini, C.; Carignani, E.; Geppi, M.; Meggiolaro, D., Light-induced formation of Pb^{3+} paramagnetic species in lead halide perovskites. *ACS Energy Lett.* **2018,** *3* (8), 1840–1847.
